# Potential survival benefits from optimized chemotherapy implementation in advanced ovarian cancer: Projections from a microsimulation model

**DOI:** 10.1371/journal.pone.0222828

**Published:** 2019-09-20

**Authors:** Anna P. Lietz, Davis T. Weaver, Alexander Melamed, Jose Alejandro Rauh-Hain, Jason D. Wright, Alexi A. Wright, Amy B. Knudsen, Pari V. Pandharipande

**Affiliations:** 1 Institute for Technology Assessment, Massachusetts General Hospital, Boston, MA, United States of America; 2 Division of Gynecologic Oncology, Vincent Obstetrics and Gynecology, Massachusetts General Hospital, Boston, MA, United States of America; 3 Gynecologic Oncology and Reproductive Medicine Department, University of Texas MD Anderson Cancer Center, Houston TX, United States of America; 4 Division of Gynecologic Oncology, Department of Obstetrics and Gynecology, Columbia University College of Physicians and Surgeons, New York, NY, United States of America; 5 Dana-Farber Cancer Institute, Boston, MA, United States of America; 6 Harvard Medical School, Boston, MA, United States of America; The Cancer Institute of New Jersey, Robert Wood Johnson Medical School, UNITED STATES

## Abstract

**Background:**

Ovarian cancer is often diagnosed in advanced stages, when survival is poor. Treatment advances have been made, but are inconsistently implemented. Our purpose was to project the maximum life expectancy gains that could be achieved in women with stage IIIC epithelial ovarian cancer if the implementation of available chemotherapy regimens could be optimized.

**Methods:**

We used a microsimulation model to estimate life expectancy benefits associated with “optimized” implementation of four post-operative chemotherapy options: standard intravenous chemotherapy; intraperitoneal + intravenous chemotherapy; bevacizumab + intravenous chemotherapy; and hyperthermic intraperitoneal chemotherapy + intravenous chemotherapy. Optimized implementation was defined as follows. Patients triaged to primary cytoreductive surgery received intraperitoneal + intravenous chemotherapy if optimally or completely cytoreduced, and bevacizumab + intravenous chemotherapy if suboptimally cytoreduced. Patients triaged to neoadjuvant chemotherapy received hyperthermic intraperitoneal chemotherapy at interval cytoreductive surgery if optimally or completely cytoreduced, and standard IV chemotherapy if suboptimally cytoreduced. Life expectancy associated with optimized implementation was compared with that of current utilization practices, estimated using published literature and the National Cancer Database. Effects of model uncertainty were evaluated in sensitivity analyses.

**Results:**

Life expectancy associated with optimized implementation vs. current practice was 76.7 vs. 64.5 months (life expectancy gain = 12.2 months). Providing intraperitoneal + intravenous chemotherapy to all eligible patients was the largest driver of life expectancy gains, due to both the potential benefit conferred by intraperitoneal + intravenous chemotherapy and the proportion of eligible women who do not receive intraperitoneal + intravenous chemotherapy in current practice.

**Conclusion:**

Population-level life expectancy in stage IIIC epithelial ovarian cancer could be substantially improved through greater uptake of available chemotherapy regimens.

## Introduction

Ovarian cancer is the 5^th^ leading cause of cancer deaths in women [1]. Most patients present with clinical symptoms at an advanced stage [2], when the disease has already spread within the peritoneal cavity [3]. The prognosis for affected patients is poor. Five-year relative survival rates for American Joint Committee on Cancer (AJCC) Stage IIIC and IV epithelial ovarian cancer (EOC)–the most common type of ovarian cancer–are 41% and 19%, respectively [4].

Treatment for advanced EOC typically includes both cytoreductive surgery and chemotherapy [5]. The decision to undergo primary cytoreductive surgery (PCS) followed by chemotherapy, or neoadjuvant chemotherapy (NACT) followed by interval cytoreductive surgery (ICS) and post-operative chemotherapy, is driven by the anticipated cytoreductive outcome at primary surgery and the patient’s risk for surgical morbidity [6]. Depending on the treatment selected (PCS vs. NACT+ICS) and the degree of cytoreduction achieved at surgery, patients are eligible for specific chemotherapy regimens that have been shown to improve survival.

Randomized control trials (RCTs) have shown that after complete or optimal cytoreduction at PCS, intraperitoneal (IP) + intravenous (IV) chemotherapy lengthens progression-free and overall survival (OS) compared to treatment with standard IV chemotherapy (carboplatin/cisplatin and paclitaxel) [7, 8]. In patients at elevated risk of progression after PCS (i.e., suboptimally cytoreduced), use of bevacizumab in addition to standard IV chemotherapy has been shown to improve OS [9–11]. In patients who receive NACT, hyperthermic intraperitoneal chemotherapy (HIPEC)–administered intraoperatively at ICS after complete or optimal cytoreduction–combined with post-operative IV chemotherapy, has been shown to lengthen recurrence-free and OS compared to standard post-operative IV chemotherapy alone [12]. While published evidence points to the potential effectiveness of these treatments, they are not currently used for all eligible patients [13–15].

In this study, we evaluated the potential life expectancy (LE) benefits of optimized implementation of standard, IP, bevacizumab, and HIPEC chemotherapy in a population of women with stage IIIC EOC. To accomplish this, we utilized a previously-developed microsimulation model for ovarian cancer treatment [16] and assigned simulated patients to the most effective treatment regimen, according to the literature, defined by whether they were initially triaged to NACT+ICS or PCS, and the level of cytoreduction achieved at surgery [6–8, 12, 17, 18]. Our purpose was to project the maximum LE gains that could be achieved in women with stage IIIC epithelial ovarian cancer (EOC) if the implementation of available chemotherapy regimens could be optimized.

## Materials and methods

We modified a previously described microsimulation model for ovarian cancer treatment [16] to study LE benefits associated with the optimized implementation of available chemotherapy regimens in stage IIIC EOC. The model was developed using TreeAge Pro Version 2017 (TreeAge Software, Williamstown, MA). The “survival” package in R version 3.4.3 was used for statistical analyses. Development of the model involved use of de-identified data from the National Cancer Database (described below); such data was used under approval from the Partners Healthcare Human Research Committee through an Institutional Review Board exemption. Additional inputs used for the model and analysis were all extracted from publicly available, de-identified data or published data.

### Overview of the model and analysis

Using the model, we compared outcomes from “optimized implementation” vs. “current practice” strategies. The “optimized implementation” strategy was recognized to be hypothetical and actively evolving. Specifically, in real-world settings, some patients are ineligible for a given chemotherapy option for reasons that cannot be changed, e.g. comorbidities. Moreover, data that inform OS associated with certain therapies in our analysis are still new (e.g., HIPEC) [12]. Other promising therapies are too new to be included (e.g., poly (ADP-ribose) polymerase (PARP) inhibitors); associated OS benefits have not yet been reported [19]. Nevertheless, our use of a microsimulation model allowed us to estimate a current, upper limit on LE benefits that could be achieved using existing chemotherapy options [7–10, 12]. In conducting this analysis, we sought to provide insight into the combined impact of current chemotherapy options, at the population level, when their implementation is optimized. We expect such information to be complementary to that gained from treatment-specific RCTs.

To project the associated LE for each strategy of interest, we modified a previously-defined standard-of-care treatment strategy within our original model [16]. In the standard-of-care strategy, patients were triaged to PCS vs. NACT+ICS based on current clinical practice, as estimated from the National Cancer Database (NCDB), which includes 70% of incident cancer cases in the US [20]. Other model parameter estimates were obtained from the NCDB [20], Centers for Disease Control and Prevention lifetables [21] and published literature [8, 10, 12, 14, 22–26]. Modifications to the original model are described below.

### Constructing the model to enable analysis by chemotherapy pursued

**Fig 1:**
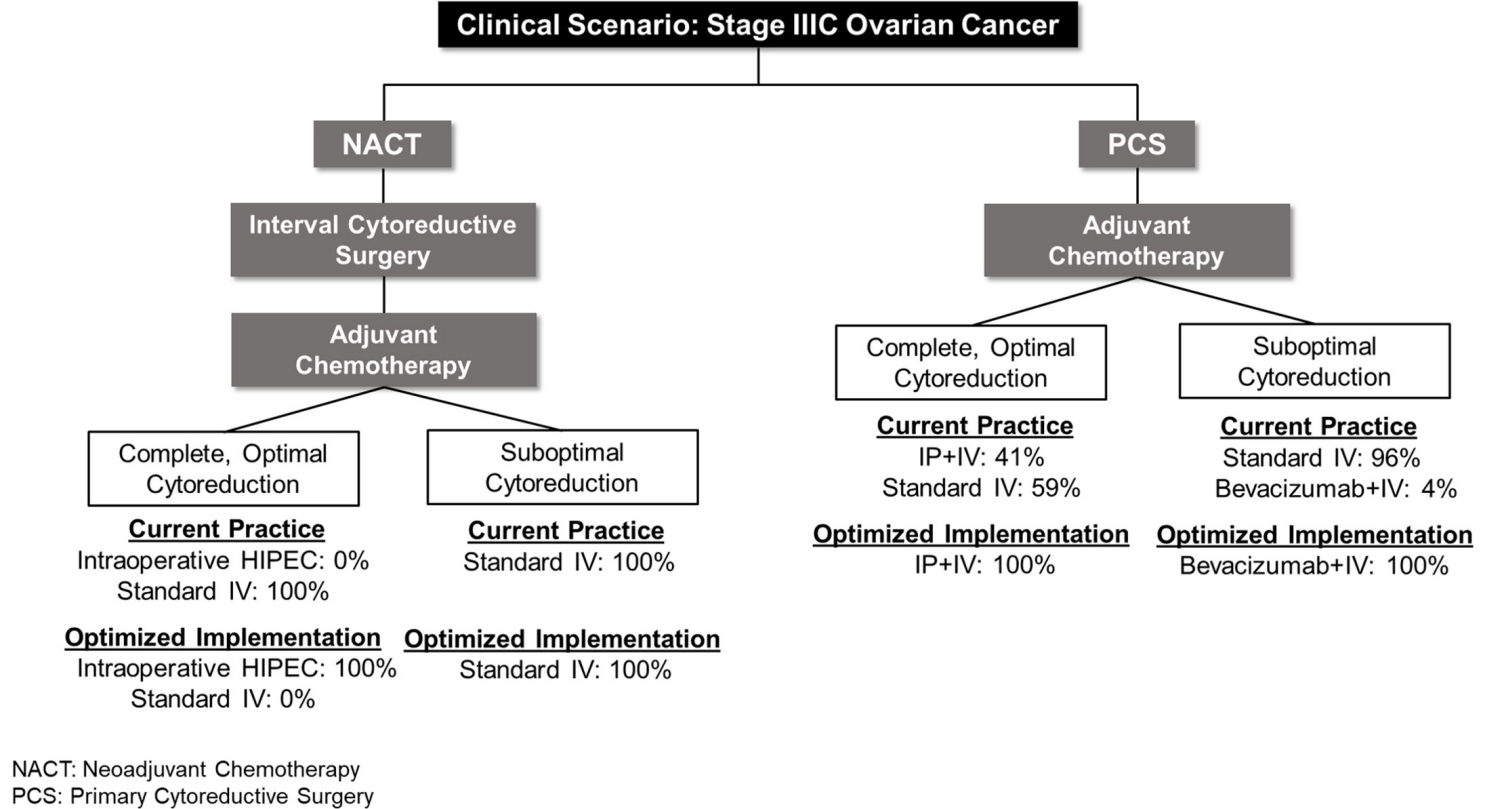
Simplified schematic of “current practice” and “optimized implementation” strategies. Chemotherapy is specified for each strategy according to treatment pursued and cytoreduction status. Probability estimates are further described in the Methods [14, 20, 26].

Fig 1 depicts chemotherapy assignments for the two principal strategies of interest in our current study: “optimized implementation” vs. “current practice.” We assumed newly-diagnosed stage IIIC EOC patients entered the model at age 63, the median age of this patient cohort [20], and underwent PCS (74.5%) or NACT+ICS (25.5%) [16, 20].

For patients triaged to PCS, three post-operative chemotherapy regimens were possible: standard IV chemotherapy; IP+IV chemotherapy; or bevacizumab+IV chemotherapy. IP+IV chemotherapy was reserved for patients who were completely (no visible disease) or optimally (visible disease <1 cm in diameter) cytoreduced [6]. Bevacizumab+IV chemotherapy was reserved for PCS patients who were suboptimally cytoreduced [9, 10, 11, 27]. Standard IV, IP+IV, and bevacizumab+IV chemotherapy utilization were incorporated in the model’s “current practice” strategy at currently observed rates (Fig 1, Table 1) [14, 26]. The survival benefit of IP+IV chemotherapy was obtained from an RCT [8]. The survival benefit of bevacizumab+IV chemotherapy was obtained from a meta-analysis combining the results of two RCTs [10].

**Table 1:** Treatment probabilities for each analyzed chemotherapy strategy.

	PCS Patients				NACT + ICS Patients		
Strategy Description	Complete/ Optimal Cytoreduction		Suboptimal Cytoreduction		Complete/ Optimal Cytoreduction		Suboptimal Cytoreduction
	IP+IV (%)	Standard IV (%)	Bevacizumab+IV (%)	Standard IV (%)	HIPEC+IV (%)	Standard IV (%)	Standard IV (%)
Current Practice (Comparison Group)	41^a^	59	4^b^	96	0	100	100
Optimized Implementation	100	0	100	0	100	0	100
Optimized IP+IV Chemotherapy	100	0	4^b^	96	0	100	100
Optimized Bevacizumab+IV Chemotherapy	41^a^	59	100	0	0	100	100
Optimized HIPEC	41^a^	59	4^b^	96	100	0	100

PCS: primary cytoreductive surgery, HIPEC: hyperthermic intraperitoneal chemotherapy, IP: intraperitoneal, NACT + ICS: neoadjuvant chemotherapy + interval cytoreductive surgery, IV: intravenous.

^a^ From Wright, AA et al.[26]

^b^ From Wright, JD et al.[14]

In some practices, dose-dense chemotherapy–defined by three doses of paclitaxel per cycle (80 mg/m^2^-1hr infusion), compared to one dose per cycle (180 mg/m^2^-3hr infusion) in standard IV chemotherapy–has also been used after a Japanese RCT demonstrated increased progression-free and OS when administered after PCS [23, 28]. However, recent US and European studies have not confirmed a survival benefit [17, 18]. As such, we assumed no survival benefit for having received dose-dense chemotherapy instead of standard IV chemotherapy. In sensitivity analysis, we explored outcomes consequent to an assumed benefit of dose-dense (over standard IV) chemotherapy [23].

For patients triaged to NACT+ICS, intraoperative HIPEC was a treatment option for patients who were completely or optimally cytoreduced at ICS. The survival benefit of HIPEC was obtained from an RCT [12]. HIPEC is not standard clinical practice, and therefore was not incorporated in the “current practice” strategy. Because HIPEC has been limited to NACT+ICS patients, and high-quality evidence is available only for this population [12], we did not include it as an option for patients triaged to PCS.

### Modeling treatment failures and survival

We modeled treatment failures and OS as described previously [16]. In brief, patients’ risk of death–whether due to stage IIIC EOC or other causes–was a function of treatment received (PCS vs. NACT+ICS), cytoreductive outcome, and receipt of full vs. partial treatment (e.g. chemotherapy completion after PCS, or ICS completion after NACT) [16]. Survival models were constructed using NCDB data for patients diagnosed with stage IIIC EOC and hazard ratios corresponding to OS estimates from published RCTs [8, 10, 12, 20, 23, 25]. After generating a reference survival curve using NCDB data for women who received NACT+ICS and achieved complete cytoreduction at ICS, we applied multiplicative, proportional hazards assumptions to adjust this curve for different treatments and cytoreductive outcomes. Hazard ratios for each chemotherapy are included in Table 2.

**Table 2:** Survival benefits of specific chemotherapy options: Hazard ratios and sensitivity analysis results.

Parameter	Base-case value^a^	Sensitivity analysis range	Reference	Life expectancy gain (months): low survival benefit	Life expectancy gain (months): high survival benefit
IV chemotherapy (HR)	1	Not varied	—	—	—
IP chemotherapy (HR)	0.75	0.59–0.97	[8]	5.6	19.1
Bevacizumab (HR)	0.85	0.74–0.96^b^	[10]	11.8	12.6
HIPEC (HR)	0.67	0.48–0.94	[12]	8.2	16.8
Time (in years) after diagnosis at which patients are assumed to be cured	12 (years)	8–16	[29]	10.6	14.6

^a^Base-case values are reported as hazard ratios for overall survival compared to standard IV chemotherapy unless otherwise specified.

^b^HR = 1 (no effect of bevacizumab) was also evaluated; see “Sensitivity Analysis” subsection of Methods and Results for further details.

HIPEC: hyperthermic intraperitoneal chemotherapy, IP: intraperitoneal chemotherapy, HR: hazard ratio.

According to estimates elicited from the NCDB (2011–2014) [20], 90.7% of stage IIIC EOC patients triaged to PCS and 82.1% of patients triaged to NACT continued to adjuvant chemotherapy or ICS, respectively. After PCS or ICS, patients were subject to 90-day surgical mortality (4.0% and 2.3%, respectively) [20]. Patients were considered cured of their disease if they survived 12 years after diagnosis [29]. Once a patient was cured, they were exposed to only age-specific all-cause mortality [21].

### Analyzing the model

Optimized chemotherapy implementation–and additional scenarios of chemotherapy selection–are described below. Using a one-month cycle length and a lifetime horizon, we predicted the mean LE and median survival among a simulated cohort of women with newly diagnosed stage IIIC EOC, under assumptions of different chemotherapy strategies. A more comprehensive description of the model and its parameters are in the published article and its online supplemental materials [16].

### Primary analyses

We compared several hypothetical, population-level treatment strategies to current clinical practice. Descriptions of each strategy and corresponding model inputs can be found below and in Table 1.

First, we compared “current practice” to “optimized implementation” (Fig 1). In the “current practice” strategy, after PCS, we assumed that 41% of eligible (i.e., completely or optimally cytoreduced) patients received IP+IV chemotherapy [26]. This estimate of 41% was derived from a multicenter study of National Comprehensive Cancer Network (NCCN) centers [26]; therefore, it may represent an overestimate when applied to the general population. In a sensitivity analysis, we evaluated the effects of varying this parameter, as further described below. Patients who were eligible but did not receive IP+IV chemotherapy received standard IV chemotherapy. Ineligible (i.e., suboptimally cytoreduced) patients received either bevacizumab+IV chemotherapy (4%) or standard IV chemotherapy [14]. After NACT and ICS, all patients received standard IV chemotherapy.

In the “optimized implementation” strategy, after PCS, all eligible patients (completely or optimally cytoreduced) received IP+IV chemotherapy. The remaining PCS patients (suboptimally cytoreduced) received bevacizumab+IV chemotherapy. All eligible NACT+ICS patients (completely or optimally cytoreduced at ICS) received HIPEC+IV chemotherapy.

Next, we evaluated three strategies in which we isolated each component of optimized chemotherapy implementation. In the “optimized IP+IV chemotherapy” strategy, we assumed all individuals who were completely or optimally cytoreduced at PCS received IP+IV chemotherapy. In the “optimized bevacizumab+IV chemotherapy” strategy, we assumed that after PCS, all suboptimally reduced patients received bevacizumab+IV chemotherapy. In the “optimized HIPEC” strategy, we assumed that after ICS, all completely or optimally cytoreduced NACT+ICS patients received HIPEC+IV chemotherapy [12]. In each of these three strategies, other than the specified assumption, chemotherapy receipt was identical to the “current practice” strategy.

Primary outcome measures, per strategy, were median survival and LE. LE was calculated by taking the average length of survival, in months, among simulated patients; median survival represented the median number of months alive among simulated patients. LE gains and improvements in median survival for “optimized” strategies were calculated by comparing with the “current practice” strategy.

### Sensitivity analysis

We conducted deterministic sensitivity analyses to evaluate the effects of uncertainty in the survival benefits associated with each chemotherapy on LE gains. We analyzed the model over the reported ranges of uncertainty (elicited from the literature) for the OS hazard ratios for IP+IV, bevacizumab+IV, and HIPEC+IV chemotherapy [8, 10, 12]. The meta-analysis utilized to inform the hazard ratio for bevacizumab in the model [10] included use of ICON7 results [11], in which non-proportional hazards were reported. To more broadly evaluate effects of uncertainty of the meta-analysis estimate, we considered an additional scenario in which there was no effect of bevacizumab (HR = 1). Additionally, we analyzed effects of uncertainty in the number of years until patients were considered cured by varying this parameter from 8 to16 years [29]. Parameter estimates and sensitivity analysis ranges are shown in Table 2.

Our estimate for IP+IV chemotherapy utilization among optimally and completely cytoreduced patients was obtained from a study among patients treated at NCCN centers [26], which may limit generalizability. To account for uncertainty in this estimate, we varied the proportion of patients in “current practice” receiving IP+IV chemotherapy after optimal or complete cytoreduction from 50–150% of the base-case value (0.21–0.62).

Recognizing that 100% adherence with the “optimized implementation” strategy was unlikely, we evaluated additional scenarios in which such implementation was imperfect. Specifically, we considered 60%, 70%, 80%, and 90% implementation of “optimized” chemotherapy. We assumed that all patients not receiving “optimized” chemotherapy would receive standard IV chemotherapy.

Lastly, we re-ran analyses of “current practice” and “optimized implementation” assuming that the survival benefit of dose-dense chemotherapy was as observed in a Japanese RCT (hazard ratio = 0.79) [23]. In the “current practice” strategy, 15.2% of all PCS patients (irrespective of cytoreductive status) received dose-dense chemotherapy [14]. In the “optimized implementation” strategy, we assumed that after PCS, all suboptimally cytoreduced patients would be given dose-dense IV chemotherapy.

## Results

### Primary analyses

**Fig 2:**
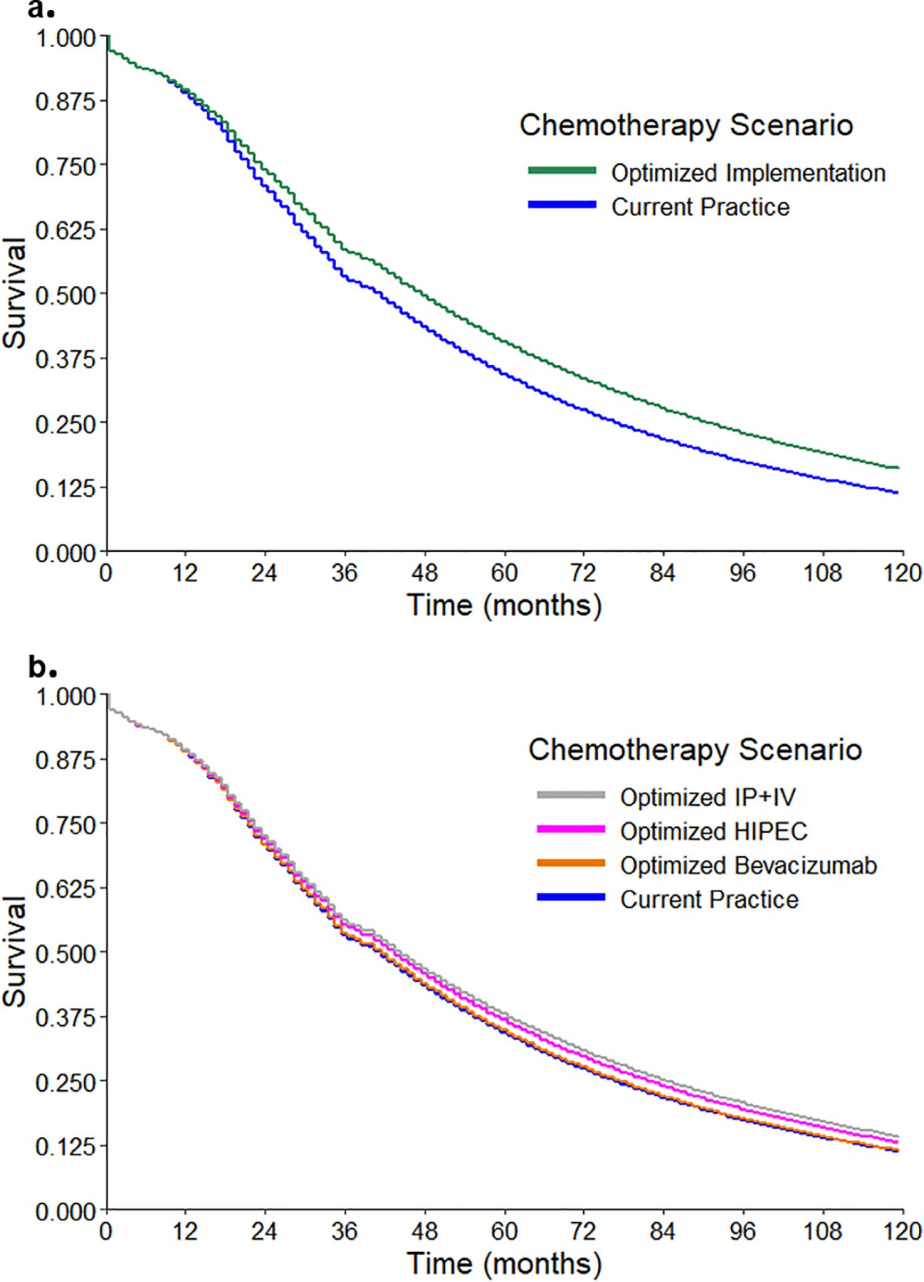
Kaplan-Meyer survival curves comparing probabilities of survival between strategies. **a) comparison of “optimized implementation” vs. “current practice.”** In “optimized implementation,” all patients who were completely or optimally cytoreduced at primary cytoreductive surgery (PCS) received intraperitoneal (IP) + intravenous (IV) chemotherapy. All patients who were suboptimally cytoreduced at PCS received bevacizumab+IV chemotherapy. All patients who were completely or optimally cytoreduced at interval cytoreductive surgery (following neoadjuvant chemotherapy) received hyperthermic intraperitoneal chemotherapy (HIPEC)+IV chemotherapy. All other patients received standard IV chemotherapy. **b) comparison of** “**optimized IP+IV”, “optimized HIPEC”, “optimized bevacizumab”, and “current practice.”** In “optimized IP+IV” all patients who were completely or optimally cytoreduced at PCS received IP+IV chemotherapy. In “optimized HIPEC” all patients who received neoadjuvant chemotherapy + interval cytoreductive surgery and had complete or optimal cytoreduction received HIPEC+IV chemotherapy. In “optimized bevacizumab” all patients who were suboptimally cytoreduced at PCS received bevacizumab+IV chemotherapy. In each of these strategies, other than the specified assumption, chemotherapy was identical to the “current practice” strategy.

We found that patients in the “current practice” strategy had a LE of 64.5 months and a median survival of 41.5 months. Patients in the “optimized implementation” strategy had a LE of 76.7 months and a median survival of 47.5 months, outperforming “current practice” by 12.2 and 6.0 months, respectively (Fig 2).

For patients in the “optimized IP+IV chemotherapy” strategy, the projected LE and median survival were 71.7 months and 44.5 months, respectively, outperforming “current practice” by 7.2 and 3.0 months, respectively.

For patients in the “optimized bevacizumab+IV chemotherapy” strategy, the projected LE and median survival were 65.0 months and 41.5 months, respectively, increasing LE by 0.5 months over “current practice”; median survival did not increase.

For patients in the “optimized HIPEC” strategy, the projected LE and median survival were 69.0 months and 43.5 months, respectively, outperforming “current practice” by 4.5 and 2.0 months, respectively.

### Sensitivity analyses

Results of sensitivity analyses pertaining to survival benefits (i.e., hazard ratio assumptions) are included in Table 2. LE gains associated with the “optimized implementation” strategy were most sensitive to the survival benefit of IP chemotherapy. When we varied the hazard ratio from the lower to the upper bound of the reported 95% confidence interval, 0.59 to 0.97 [8], LE gains varied from 19.1 to 5.6 months. Importantly, when we varied the proportion of patients receiving IP+IV chemotherapy in “current practice” from 50–150% of the base-case estimate, we found that LE gains associated with “optimized implementation” varied from 9.7 to 14.6 months. LE gains were moderately sensitive to the survival benefit of HIPEC. When we varied the hazard ratio from the lower to the upper bound of the reported 95% confidence interval, 0.48 to 0.94 [12], LE gains varied from 16.8 to 8.2 months.

LE gains associated with the “optimized implementation” strategy were relatively insensitive to the survival benefit of bevacizumab. When we varied the hazard ratio from the lower to the upper bound of the reported 95% confidence interval, 0.74 to 0.96 [10], LE gains varied from 12.6 to 11.8 months (Table 2). Furthermore, when we assumed no survival benefit associated with bevacizumab + IV chemotherapy, the LE gain associated with “optimized implementation” was 11.7 months. LE gains were also relatively insensitive to year of assumed cure. When we varied this parameter from 8 to 16 years, LE gains varied from 14.6 to 10.6 months. Lastly, when we assumed that dose-dense chemotherapy afforded a survival benefit, our results did not change substantially; LE associated with “current practice” was estimated to be 66.1 months, and with “optimized implementation” was estimated to be 76.9 months (LE gain = 10.8 months vs.12.2 months in the primary analysis).

As the proportion of eligible patients who were subject to “optimized implementation” increased, LE gains increased (Table 3). For example, at 60% implementation, the LE gain associated with “optimized” chemotherapy was 5.3 months, whereas at 90% implementation it was 10.4 months (Table 3).

**Table 3:** Life expectancy (LE) associated with imperfect “optimized implementation”.

	LE, in months (difference from current practice)	Median survival, in months (difference from current practice)
Current Practice	64.5 (0)	41.5 (0)
60% Optimized	69.8 (+5.3)	43.5 (+2)
70% Optimized	71.5 (+7.0)	44.5 (+3)
80% Optimized	73.2 (+8.7)	45.5 (+4)
90% Optimized	74.9 (+10.4)	46.5 (+5)

## Discussion

RCTs studying IP+IV, bevacizumab, and HIPEC chemotherapy options have demonstrated a survival benefit–relative to standard IV chemotherapy–for specific subpopulations of women with advanced EOC [7–9, 12]. Even so, these regimens are not being used in all eligible patients [14, 26]. We estimated that optimized implementation of these chemotherapy regimens could improve population-level LE in women with stage IIIC ovarian cancer by up to 12.2 months. Among these regimens, we found that that increasing IP chemotherapy may provide the greatest LE benefit (7.2 months). This result is driven by the potential survival benefit associated with IP+IV chemotherapy and by the large proportion of eligible PCS patients (optimal or complete cytoreduction) who do not currently receive this treatment. In contrast, optimizing use of bevacizumab in suboptimally cytoreduced PCS patients affects a much smaller proportion of patients (~11% of PCS patients) and confers a lesser survival benefit.

The gap between who is eligible and who receives IP chemotherapy is not well understood [13, 26]. In part, it exists because some patients cannot tolerate IP chemotherapy. A study performed at Memorial Sloan-Kettering Cancer Center reported that from 2006–2013, 79% of eligible stage III ovarian, fallopian, or primary peritoneal cancer patients received IP chemotherapy, supporting the notion that its use could be increased among eligible patients [13]. However, this study also reported that addressing modifiable factors may have allowed this proportion to increase to 88% at maximum; the remaining 12% patients had unmodifiable factors [13]. This gap is also likely due to physicians’ uncertainty about the underlying effectiveness of IP chemotherapy. The results of a recent RCT (GOG-252), in which IP+bevacizumab did not confer increased progression-free survival relative to IV+bevacizumab, raise questions about the true benefit of IP chemotherapy [30]. Because OS estimates remain unreported, we could not incorporate the results of this trial into our analysis [30]. Nevertheless, through an analysis focused on optimizing IP+IV chemotherapy implementation in isolation–and through sensitivity analysis–our study provides valuable insight into the potential contribution of IP+IV chemotherapy to LE gains over a wide range of assumptions about its effectiveness.

Our study has additional limitations that merit mention. First, the literature regarding the clinical benefit of dose-dense IV chemotherapy administration is conflicting [17, 18, 23, 28]. However, we found that practice differences in the administration of dose-dense IV chemotherapy administration were not the primary driver of our results. Second, approval for the use of bevacizumab after PCS occurred within the past 1–2 years; current utilization rates are therefore difficult to determine. Even so, the effects of bevacizumab utilization on our results were modest, due to the limited survival benefits and the small proportion of women eligible. Third, our model does not include maintenance treatment with poly (ADP-ribose) polymerase (PARP) inhibitors. A recently published RCT found that use of PARP inhibitors as first-line therapy significantly increased progression-free survival in late-stage ovarian cancer patients with a somatic or germline BRCA 1/2 mutation [19]. The trial, however, has not yet definitively shown an increase in OS [19], thus we did not incorporate treatment with PARP inhibitors into the model. Nevertheless, for the approximately 20–25% of ovarian cancer patients with a germline or somatic BRCA 1/2 mutation [31], PARP inhibitors show promise. Fourth, assumed survival benefits associated with each chemotherapy regimen come from RCTs with an inherent bias for healthier patients; such benefits are not likely to be generalizable to the entire population.

Lastly, due to the absence of robust data, we did not simulate the impact of the various chemotherapy options on patients’ quality-of-life. While there is a difference in overall quality-of-life scores between IP+IV vs. standard IV chemotherapy during and in the few months after treatment (IP+IV scores are lower), after one year there is no reported difference [8]. Similarly, while there is a difference between overall quality-of-life scores at baseline and during HIPEC+IV treatment for peritoneal carcinomatosis (lower on-treatment scores), there is no reported difference after one year [32]. It should also be noted that our analyses are intended to explore population-level, and not patient-level, outcomes. Treatment decisions for individual patients should account for more granular factors such as age, comorbidities, treatment preferences, and surgical and center-level expertise.

## Conclusion

Despite these limitations, our results provide valuable context for clinicians and researchers in this field. We found that survival in a population of women diagnosed with stage IIIC EOC may be substantially improved when chemotherapy receipt is optimized, underscoring the importance of overcoming barriers to the use of optimal therapies. Equally, in an era of new and evolving treatment approaches for ovarian cancer, our study provides an example of how simulation models can be used to rapidly integrate new data on treatment effectiveness as it emerges, providing unique insight into the population-level impact of new therapies in the coming years.

## Supporting information

S1 File “Current practice” with baseline IP at 150%. Model output for life expectancy under “current practice” strategy assuming baseline proportion of eligible patients recieving IP chemotherapy is 0.615.
(TXT)

S2 File “Current practice” with baseline IP at 50%. Model output for life expectancy under “current practice” strategy assuming baseline proportion of eligible patients recieving IP chemotherapy is 0.205.
(TXT)

S3 File “Current practice” assuming cure at 16 years. Model output for life expectancy under “current practice” strategy assuming that patients are cured after surviving 16 years from diagnosis.
(TXT)

S4 File “Current practice” assuming cure at 8 years. Model output for life expectancy under “current practice” strategy assuming that patients are cured after surviving 8 years from diagnosis.
(TXT)

S5 File “Current practice”. Model output for life expectancy under the “current practice” strategy.
(TXT)

S6 File “Current practice” with a high hazard ratio on bevacizumab. Model output for life expectancy under the “current practice” strategy with a hazard ratio of 0.96 on bevacizumab+IV.
(TXT)

S7 File “Current practice” with a high hazard ratio on HIPEC. Model output for life expectancy under the “current practice” strategy with a hazard ratio of 0.94 on HIPEC+IV.
(TXT)

S8 File “Current practice” with a high hazard ratio on IP. Model output for life expectancy under the “current practice” strategy with a hazard ratio of 0.97 on IP+IV.
(TXT)

S9 File “Current practice” with a low hazard ratio on bevacizumab. Model output for life expectancy under the “current practice” strategy with a hazard ratio of 0.74 on bevacizumab+IV.
(TXT)

S10 File “Current practice” with a low hazard ratio on HIPEC. Model output for life expectancy under the “current practice” strategy with a hazard ratio of 0.48 on HIPEC+IV.
(TXT)

S11 File “Current practice” with a low hazard ratio on IP. Model output for life expectancy under the “current practice” strategy with a hazard ratio of 0.59 on IP+IV.
(TXT)

S12 File “Current practice” assuming no benefit from bevacizumab. Model output for life expectancy under the “current practice” strategy assuming that bevacizumab has no additional survivial benefit (HR = 1.0) compared to standard IV chemotherapy.
(TXT)

S13 File “Current practice” assuming use and a survival benefit of dose dense chemotherapy. Model output for life expectancy under the “current practice” strategy assuming that dose dense is used on 15.2% of all PCS patients and that it confers a survival benefit (HR = 0.79).
(TXT)

S14 File “Optimized implementation” at 60%. Model output for life expectancy under the “optimized implementation” strategy assuming that only 60% of eligible patients receive the optimized therapy according to their cytoreductive status. Patients who do not receive the optimal therapy are assumed to receive standard IV chemotherapy.
(TXT)

S15 File “Optimized implementation” at 70%. Model output for life expectancy under the “optimized implementation” strategy assuming that only 70% of eligible patients receive the optimal therapy according to their cytoreductive status. Patients who do not receive the optimal therapy are assumed to receive standard IV chemotherapy.
(TXT)

S16 File “Optimized implementation” at 80%. Model output for life expectancy under the “optimized implementation” strategy assuming that only 80% of eligible patients receive the optimal therapy according to their cytoreductive status. Patients who do not receive the optimal therapy are assumed to receive standard IV chemotherapy.
(TXT)

S17 File “Optimized implementation” at 90%. Model output for life expectancy under the “optimized implementaion” strategy assuming that only 90% of eligible patients receive the optimal therapy according to their cytoreductive status. Patients who do not receive the optimal therapy are assumed to receive standard IV chemotherapy.
(TXT)

S18 File “Optimized implementation” of bevacizumab. Life expectancy output for strategy in which all primary cytoreductive sugery patients who are suboptimally cytoreduced receive bevacizumab+IV chemotherapy. All other patients receive chemotherapy according to “current practice”.
(TXT)

S19 File “Optimized implementation” assuming cure at 16 years. Model output for life expectancy under “optimized implementation” strategy assuming that patients are cured after surviving 16 years from diagnosis.
(TXT)

S20 File “Optimized implementation” assuming cure at 8 years. Model output for life expectancy under the “optimized implementation” strategy assuming that patients are cured 8 years from diagnosis.
(TXT)

S21 File “Optimized implementation” with a high hazard ratio on bevacizumab. Model output for life expectancy under the “optimized implementation” strategy with a hazard ratio of 0.96 on bevacizumab+IV.
(TXT)

S22 File “Optimized implementation” with a high hazard ratio on HIPEC. Model output for life expectancy under the “optimized implementation” strategy with a hazard ratio of 0.94 on HIPEC+IV.
(TXT)

S23 File “Optimized implementation” with a high hazard ratio on IP. Model output for life expectancy under the “optimized implementation” strategy with a hazard ratio of 0.97 on IP+IV.
(TXT)

S24 File “Optimized implementation” of HIPEC. Life expectancy output assuming that 100% of complete or optimally cytoreduced patients who received neoadjuvant chemotherapy and interval cytoreductive surgery are given HIPEC+IV.
(TXT)

S25 File “Optimized implementation”. Life expectancy output under the “optimized implementation” strategy.
(TXT)

S26 File “Optimized implementation” of IP+IV. Life expectancy output assuming that 100% of all primary cytoreductive surgery patients who are complete or optimally cyroreduced receive IP+IV chemotherapy.
(TXT)

S27 File “Optimized implementation” with a low hazard ratio on bevacizumab. Model output for life expectancy under the “optimized implementation” strategy with a hazard ratio of 0.74 on bevacizumab+IV.
(TXT)

S28 File “Optimized implementation” a low hazard ratio on HIPEC. Model output for life expectancy under the “optimized implementation” strategy with a hazard ratio of 0.48 on HIPEC.
(TXT)

S29 File “Optimized implementation” a low hazard ratio on IP. Model output for life expectancy under the “optimized implementation” strategy with a hazard ratio of 0.59 on IP+IV.
(TXT)

S30 File “Optimized implementation” assuming no benefit from bevacizumab. Model output for life expectancy under the “optimized implementation” strategy assuming that bevacizumab has no additional survivial benefit (HR = 1.0) compared to standard IV chemotherapy.
(TXT)

S31 File “Optimized implementation” assuming use and a survival benefit of dose dense chemotherapy. Model output for life expectancy under the “optimized implementation” strategy assuming that dose dense is used on all primary cytoreductive surgery patients who were suboptimally cytoreduced and that it confers a survival benefit (HR = 0.79).
(TXT)

## References

[1] American Cancer Society. Cancer Facts & Figures. Atlanta, GA, 2019.

[2] Siegel RL, Miller KD, Jemal A. Cancer statistics, 2018. CA Cancer J Clin. 2018;68(1):7–30. Epub 2018/01/10. 10.3322/caac.21442 . 29313949

[3] American Cancer Society. Ovarian cancer stages 2018 [cited 2018 December]. Available from: https://www.cancer.org/cancer/ovarian-cancer/detection-diagnosis-staging/staging.html.

[4] American Cancer Society. Survival Rates for Ovarian Cancer by Stage 2018 [cited 2018 December]. Available from: https://www.cancer.org/cancer/ovarian-cancer/detection-diagnosis-staging/survival-rates.html.

[5] Coleman RL, Monk BJ, Sood AK, Herzog TJ. Latest research and treatment of advanced-stage epithelial ovarian cancer. Nat Rev Clin Oncol. 2013;10(4):211–24. 10.1038/nrclinonc.2013.5 23381004 PMC3786558

[6] Wright AA, Bohlke K, Armstrong DK, Bookman MA, Cliby WA, Coleman RL, et al Neoadjuvant chemotherapy for newly diagnosed, advanced ovarian cancer: Society of Gynecologic Oncology and American Society of Clinical Oncology Clinical Practice Guideline. Gynecol Oncol. 2016;143(1):3–15. 10.1016/j.ygyno.2016.05.022 27650684 PMC5413203

[7] Alberts DS, Liu PY, Hannigan EV, O'Toole R, Williams SD, Young JA, et al Intraperitoneal cisplatin plus intravenous cyclophosphamide versus intravenous cisplatin plus intravenous cyclophosphamide for stage III ovarian cancer. N Engl J Med. 1996;335(26):1950–5. 10.1056/NEJM199612263352603 . 8960474

[8] Armstrong DK, Bundy B, Wenzel L, Huang HQ, Baergen R, Lele S, et al Intraperitoneal cisplatin and paclitaxel in ovarian cancer. N Engl J Med. 2006;354(1):34–43. 10.1056/NEJMoa052985 . 16394300

[9] Perren TJ, Swart AM, Pfisterer J, Ledermann JA, Pujade-Lauraine E, Kristensen G, et al A phase 3 trial of bevacizumab in ovarian cancer. N Engl J Med. 2011;365(26):2484–96. 10.1056/NEJMoa1103799 . 22204725

[10] Wu YS, Shui L, Shen D, Chen X. Bevacizumab combined with chemotherapy for ovarian cancer: an updated systematic review and meta-analysis of randomized controlled trials. Oncotarget. 2017;8(6):10703–13. 10.18632/oncotarget.12926 27793044 PMC5354693

[11] Oza AM, Cook AD, Pfisterer J, Embleton A, Ledermann JA, Pujade-Lauraine E, et al Standard chemotherapy with or without bevacizumab for women with newly diagnosed ovarian cancer (ICON7): overall survival results of a phase 3 randomised trial. Lancet Oncol. 2015;16(8):928–36. 10.1016/S1470-2045(15)00086-8 26115797 PMC4648090

[12] van Driel WJ, Koole SN, Sikorska K, Schagen van Leeuwen JH, Schreuder HWR, Hermans RHM, et al Hyperthermic Intraperitoneal Chemotherapy in Ovarian Cancer. N Engl J Med. 2018;378(3):230–40. 10.1056/NEJMoa1708618 . 29342393

[13] Schlappe BA, Mueller JJ, Zivanovic O, Gardner GJ, Long Roche K, Sonoda Y, et al Cited rationale for variance in the use of primary intraperitoneal chemotherapy following optimal cytoreduction for stage III ovarian carcinoma at a high intraperitoneal chemotherapy utilization center. Gynecol Oncol. 2016;142(1):13–8. 10.1016/j.ygyno.2016.05.015 27189456 PMC4917455

[14] Wright JD, Hou JY, Burke WM, Tergas AI, Chen L, Hu JC, et al Utilization and Toxicity of Alternative Delivery Methods of Adjuvant Chemotherapy for Ovarian Cancer. Obstet Gynecol. 2016;127(6):985–91. 10.1097/AOG.0000000000001436 27159764 PMC4879086

[15] Melamed A, Hinchcliff EM, Clemmer JT, Bregar AJ, Uppal S, Bostock I, et al Trends in the use of neoadjuvant chemotherapy for advanced ovarian cancer in the United States. Gynecol Oncol. 2016;143(2):236–40. 10.1016/j.ygyno.2016.09.002 . 27612977

[16] Weaver DT, Raphel TJ, Melamed A, Rauh-Hain JA, Schorge JO, Knudsen AB, et al Modeling treatment outcomes for patients with advanced ovarian cancer: Projected benefits of a test to optimize treatment selection. Gynecol Oncol. 2018;149(2):256–262. 10.1016/j.ygyno.2018.02.007 . 29486993

[17] Clamp AR, McNeish I, Dean A, Gallardo D, Weon-Kim J, O'Donnell D, et al 929O_PRICON8: A GCIG phase III randomised trial evaluating weekly dose- dense chemotherapy integration in first-line epithelial ovarian/fallopian tube/primary peritoneal carcinoma (EOC) treatment: Results of primary progression- free survival (PFS) analysis. Annals of Oncology. 2017;28(suppl_5):mdx440.039–mdx440.039. 10.1093/annonc/mdx440.039

[18] Chan JK, Brady MF, Penson RT, Huang H, Birrer MJ, Walker JL, et al Weekly vs. Every-3-Week Paclitaxel and Carboplatin for Ovarian Cancer. N Engl J Med. 2016;374(8):738–48. 10.1056/NEJMoa1505067 26933849 PMC5081077

[19] Moore K, Colombo N, Scambia G, Kim BG, Oaknin A, Friedlander M, et al Maintenance Olaparib in Patients with Newly Diagnosed Advanced Ovarian Cancer. N Engl J Med. 2018 10.1056/NEJMoa1810858 . 30345884

[20] Bilimoria KY, Stewart AK, Winchester DP, Ko CY. The National Cancer Data Base: a powerful initiative to improve cancer care in the United States. Ann Surg Oncol. 2008;15(3):683–90. 10.1245/s10434-007-9747-3 18183467 PMC2234447

[21] Centers for Disease Control, National Center for Health Statistics. United States Life Tables. 2011. 26460931

[22] Bookman MA, Brady MF, McGuire WP, Harper PG, Alberts DS, Friedlander M, et al Evaluation of new platinum-based treatment regimens in advanced-stage ovarian cancer: a Phase III Trial of the Gynecologic Cancer Intergroup. J Clin Oncol. 2009;27(9):1419–25. 10.1200/JCO.2008.19.1684 19224846 PMC2668552

[23] Katsumata N, Yasuda M, Isonishi S, Takahashi F, Michimae H, Kimura E, et al Long-term results of dose-dense paclitaxel and carboplatin versus conventional paclitaxel and carboplatin for treatment of advanced epithelial ovarian, fallopian tube, or primary peritoneal cancer (JGOG 3016): a randomised, controlled, open-label trial. Lancet Oncol. 2013;14(10):1020–6. 10.1016/S1470-2045(13)70363-2 . 23948349

[24] Kehoe S, Hook J, Nankivell M, Jayson GC, Kitchener H, Lopes T, et al Primary chemotherapy versus primary surgery for newly diagnosed advanced ovarian cancer (CHORUS): an open-label, randomised, controlled, non-inferiority trial. Lancet. 2015;386(9990):249–57. 10.1016/S0140-6736(14)62223-6 . 26002111

[25] Vergote I, Trope CG, Amant F, Kristensen GB, Ehlen T, Johnson N, et al Neoadjuvant chemotherapy or primary surgery in stage IIIC or IV ovarian cancer. N Engl J Med. 2010;363(10):943–53. 10.1056/NEJMoa0908806 . 20818904

[26] Wright AA, Cronin A, Milne DE, Bookman MA, Burger RA, Cohn DE, et al Use and Effectiveness of Intraperitoneal Chemotherapy for Treatment of Ovarian Cancer. J Clin Oncol. 2015;33(26):2841–7. 10.1200/JCO.2015.61.4776 26240233 PMC4554746

[27] Burger RA, Brady MF, Bookman MA, Fleming GF, Monk BJ, Huang H, et al Incorporation of bevacizumab in the primary treatment of ovarian cancer. N Engl J Med. 2011;365(26):2473–83. 10.1056/NEJMoa1104390 . 22204724

[28] Katsumata N, Yasuda M, Takahashi F, Isonishi S, Jobo T, Aoki D, et al Dose-dense paclitaxel once a week in combination with carboplatin every 3 weeks for advanced ovarian cancer: a phase 3, open-label, randomised controlled trial. Lancet. 2009;374(9698):1331–8. 10.1016/S0140-6736(09)61157-0 19767092

[29] McLaughlin JR, Rosen B, Moody J, Pal T, Fan I, Shaw PA, et al Long-term ovarian cancer survival associated with mutation in BRCA1 or BRCA2. J Natl Cancer Inst. 2013;105(2):141–8. 10.1093/jnci/djs494 23257159 PMC3611851

[30] Walker JL, Brady MF, Wenzel L, Fleming GF, Huang HQ, DiSilvestro PA, et al Randomized Trial of Intravenous Versus Intraperitoneal Chemotherapy Plus Bevacizumab in Advanced Ovarian Carcinoma: An NRG Oncology/Gynecologic Oncology Group Study. J Clin Oncol. 2019:JCO1801568. 10.1200/JCO.18.01568 . 31002578 PMC6544459

[31] Frey MK, Pothuri B. Homologous recombination deficiency (HRD) testing in ovarian cancer clinical practice: a review of the literature. Gynecol Oncol Res Pract. 2017;4:4 10.1186/s40661-017-0039-8 28250960 PMC5322589

[32] Passot G, Bakrin N, Roux AS, Vaudoyer D, Gilly FN, Glehen O, et al Quality of life after cytoreductive surgery plus hyperthermic intraperitoneal chemotherapy: a prospective study of 216 patients. Eur J Surg Oncol. 2014;40(5):529–35. 10.1016/j.ejso.2013.11.019 . 24370285

